# Editorial: Cellular dormancy—State determination and plasticity

**DOI:** 10.3389/fcell.2022.984347

**Published:** 2022-08-04

**Authors:** Guang Yao, Jyotsna Dhawan, Alexis R. Barr

**Affiliations:** ^1^ Department of Molecular and Cellular Biology, Tucson, AZ, United States; ^2^ Centre for Cellular & Molecular Biology (CCMB), Hyderabad, Andhra Pradesh, India; ^3^ MRC London Institute of Medical Sciences (LMS), London, England, United Kingdom

**Keywords:** cellular dormancy, quiescence, senescence, heterogeneity, plasticity

## Introduction

Most cells in the adult human body are non-dividing. Among these dormant cells, many are postmitotic and permanently out of the cell cycle (e.g., terminally differentiated and senescent cells), while a small sub-population, namely quiescent cells (such as adult stem and progenitor cells), can re-enter the cell cycle and divide in response to physiological cues. The balance between cell proliferation and dormancy plays critical roles in tissue homeostasis, repair, regeneration, and development. Pathological changes in cellular dormancy can lead to a wide range of hyper- or hypo-proliferation diseases, including cancer, fibrosis, autoimmunity, anemia, and aging.

Cellular dormancy exhibits significant heterogeneity and plasticity. Like sleep that has shallow and deep stages, dormancy appears to have different depths that are inversely correlated with the likelihood of cell cycle re-entry upon stimulation (Coller et al., 2006; Rodgers et al., 2014; Kwon et al., 2017). We lack a fundamental understanding of what determines and shapes cellular dormancy states: What cellular activities control dormancy entry, maintenance, and exit? What physiological signals regulate cell dormancy in different tissues and organs? Can we manipulate dormant states to improve human health, e.g., against aging and cancer?

This Research Topic includes original research and review articles that address the fascinating and complex states of cell dormancy in both human and model organisms. Three broad themes are collectively addressed:

## Dormancy entry, maintenance, and exit

The proliferative cycle can be described by four consecutive phases: G1, S (DNA synthesis), G2, and M (Mitosis). Dormant cells sit outside of this proliferative cycle, with quiescent cells still retaining proliferative potential but senescent and terminally differentiated cells losing it. While the field has studied and revealed a lot about the successive cell cycle phase transitions, we have much to learn regarding how cells enter, maintain and, in the case of quiescent cells, exit, dormant states.

The review by Brookes describes advances in our understanding of how cells exit quiescence back into the proliferative cell cycle. The time taken for cells to exit quiescence is highly variable and this review integrates and discusses a flurry of recent single-cell imaging studies that have started to change the way we think about how cells exit dormancy.

It has been tempting to speculate that epigenetic mechanisms may play an important role in the control of cell dormancy, but the specifics have been hard to pin down. The review by Bonitto et al. discussed the histone code hypothesis enabling reversible arrest. Given the technical advances, and new appreciation of cooperative nuclear events, understanding chromatin conformational dynamics is likely to help elucidate the control of transitions into and out of dormancy.

It is becoming clear that the signalling pathways coordinating dormancy entry and exit differ between cell and tissue types. Here, two review articles highlight these differences in two systems: in CD8^+^ T-cells by Lewis and Ly and in the nervous system by Nandakumar et al. We anticipate that this will be a burgeoning field in the coming years where we try to understand the commonalities, but also the key cell type-specific differences. This could have important implications for our understanding for how dysregulated cell cycle components contribute to tissue-specific tumours. On the subject of cancer, to round up this set of articles, a research article by Pulianmackal et al. investigates asynchronous proliferation-quiescence decisions in pairs of daughters after mitosis in the context of prostate cancer - how it might arise and how this could contribute to tumor heterogeneity.

## Dormancy regulation at the tissue and organismal levels

Beyond the cellular level, higher order mechanisms are required to coordinate, maintain or impose cell dormancy in the organism. Three articles in this Research Topic highlight such high order mechanisms in development and evolution.

Body-wide changes across different cell and tissue types can be mapped to hormonal fluctuations. However, cell-type specificity of dormancy-regulation pathways needs more explanation. Li et al. report that a Ca^2+^-dependent secreted signal Stc1a (glycoprotein) in zebrafish suppresses local IGF-Akt-mTOR signaling and promotes the quiescence of Ca^2+^-sensitive epithelial cells (ionocytes). Ionocytes functionally resemble mammalian renal epithelia, suggesting a potential role of Ca^2+^-dependent pathways in regulating renal cell quiescence, with implications for kidney disease.

Cells *in vivo* experience interstitial fluid flows whose effects on cell dormancy are little known. By modeling cellular behaviors in microfluidic chambers, Liu et al. showed a faster extracellular flow drives cells to a more shallow quiescence, by increasing shear stress and extracellular factor replacement. This finding may help us understand how physico-chemical cues affect heterogeneous quiescence-proliferation balance *in situ*, impacting tissue dynamics.

A comprehensive review by van der Weijden and Bulut-Karslioglu addresses the connection between diapause, a reversible pause of early embryonic development, and the maintenance of potency and dormancy in adult stem cells. They survey diapause in a number of species and discuss body-wide signals at different diapause stages for dormancy induction, maintenance, and exit. Overall, this review identifies gaps in knowledge of dormant embryo and adult stem cells, understanding of which could permit interventions in diseases where quiescence is pathologically altered.

## Dormancy, diseases, and evolution

It is important to understand dormancy in the context of disease. Senescent cells accumulate during aging and in response to chemo- and radio-therapy in cancer treatment. Senescent cells contribute to aging processes and may promote tumorigenesis, and thus efforts are directed towards trying to eliminate senescent cells. He et al. discuss the challenges and ways forward in generating new senolytic treatments, particularly, how to target the increased apoptotic resistance of senescent cells.

As well as senescence, dormant cells contribute to cancer progression in other ways. For example, dormant cells are more resistant to chemotherapy than proliferating cells, and tumor cells can migrate from the primary tumor and lodge at metastatic sites in dormant states before reawakening years, or even decades, later, driving tumor relapse. Wiecek et al. tackles the question of which mutational processes give rise to dormant cells in tumors, as a route to start to understand how to tackle tumor dormancy and improve patient outcomes. By taking the vast amount of data from the TCGA database and profiling tumors across cancer types for signatures of tumor dormancy, they identify an intriguing link between tumor dormancy and APOBEC-mediated mutagenesis.

Finally, Daignan-Fornier et al. discusses how quiescent states that emerged in unicellular organisms as a survival mechanism may have been adapted as multicellular organisms emerged. In this regard, quiescence may have co-evolved with and acted as a mechanism to promote cell specialization across different cell and tissue types.

Together, the articles in this Research Topic cover features and implications of cellular dormancy states in evolution, development and disease, and highlight the gaps that remain for a more mechanistic understanding.
